# Prevalence of HMTV in breast carcinomas and unaffected tissue from Mexican women

**DOI:** 10.1186/1471-2407-14-942

**Published:** 2014-12-12

**Authors:** Alberto Cedro-Tanda, Alejandro Córdova-Solis, Teresa Juárez-Cedillo, Emmanuel Pina-Jiménez, Marta E Hernández-Caballero, Christian Moctezuma-Meza, Germán Castelazo-Rico, Alejandro Gómez-Delgado, Alejandro Cruz Monsalvo-Reyes, Fabio A Salamanca-Gómez, Diego J Arenas-Aranda, Normand García-Hernández

**Affiliations:** Medical Research Unit in Human Genetics, Pediatric Hospital, CMN S-XXI, IMSS, Mexico City, Mexico; Biological Sciences Program, UNAM, Mexico City, Mexico; Biomedical Sciences Program, UNAM, Mexico City, Mexico; Epidemiologic and Health Service Research Unit, Aging Area, CMN S-XXI, IMSS, Mexico City, Mexico; School of Medicine, Polytechnic National Institute, Mexico City, Mexico; UMAE, Gynecology and Obstetrics Hospital No. 3. CMN “La Raza”, Mexico City, Mexico; Medical Research Unit in Infectious and Parasitic Diseases, CMN S-XXI, IMSS, Mexico City, Mexico; Sequencing Unit Department, Molecular Biochemistry, UBIPRO, FES-Iztacala, UNAM, Mexico City, Mexico; Unidad de Investigación Médica en Genética Humana, Hospital de Pediatría, 2do Piso, CMN S-XXI, Av. Cuauhtémoc 330. Col. Doctores, Mexico City, CP 06720, México

**Keywords:** Breast cancer, HMTV, MMTV, Human mammary tumor virus, Mouse mammary tumor virus, Viral oncogenesis

## Abstract

**Background:**

Breast cancer is a complex multifactorial genetic disease. Among other factors, race and, to an even greater extent, viruses are known to influence the development of this heterogeneous disease. It has been reported that MMTV-like (HMTV) gene sequences with a 90 to 98% homology to mouse mammary tumor virus are found in several populations with a prevalence range of 0 to 74%. In the Mexican population, 4.2% of patients with breast cancer exhibit the presence of HMTV (MMTV-like) sequences. The aim of this study was to evaluate the presence and current prevalence of retroviral HMTV (MMTV-like) sequences in breast cancer in Mexican women.

**Methods:**

We used nested PCR and real-time PCR with a TaqMan probe. As a positive control, we used the C3H MMTV strain inserted into pBR322 plasmid. To confirm that we had identified the HMTV sequences, we sequenced the amplicons and compared these sequences with those of MMTV and HMTV (GenBank AF033807 and AF346816).

**Results:**

A total of 12.4% of breast tumors were HMTV-positive, and 15.7% of the unaffected tissue samples from 458 patients were HMTV-positive. A total of 8.3% of the patients had both HMTV-positive tumor and adjacent tissues. The HMTV-positive samples presented 98% similarity to the reported HMTV sequence.

**Conclusions:**

These results confirm that the HMTV sequence is present in breast tumors and non-affected tissues in the Mexican population. HMTV should be considered a prominent causative agent of breast cancer.

## Background

Breast cancer (BC) is one of the most prevalent malignancies in women worldwide, but the etiology of this disease remains unclear and continues to be a health problem as the leading cause of cancer death among women [1–3]. Many studies indicate that this disease is complex and multifactorial, and there are many risk factors that promote tumor development, such as age, parity, hormonal influence, diet, environment, geographic location, a race and inherited genetic predisposition [4–6]. However, the molecular mechanisms related to breast carcinogenesis remain poorly understood, particularly because of the biological heterogeneity of the disease [3].

BC is a multistep disease, and viral infection may play a role in one or more of the steps in its pathogenesis [7–11]. This hypothesis is based on the proven role of mouse mammary tumor virus (MMTV) as the causal agent of mammary tumors in mice [12–14]. Subsequently, various investigators have shown that direct infection with MMTV can infect human cells [9, 15–18].

Some studies have indicated that a virus similar to MMTV, namely MMTV-like virus, which is also known as human mammary tumor virus (HMTV), may be a risk factor for human BC [10, 11].

Several studies have demonstrated the presence of the MMTV-like *env* gene sequence with prevalences in the range of 0 to 74% of BC cases in several countries, including prevalences of 0% in Japan [19], Austria [20] and the United Kingdom [21], 0.8% in Vietnam [17], 17% in China [22], 31% in Argentina [17], 38% in Italy [17], 40% in United States [23], 42% in Australia [11], and 74% in Tunisia [17]. Mexico has reported a prevalence of 4.2% [24]; thus, these data remain controversial. However, other researchers have not been able to reproduce the same results, which is attributed to the use of a different methodology to identify the virus [25].

Pogo *et al.* [10] reported that inflammatory breast cancer in American women shows a higher incidence of viral sequences (71%) than sporadic breast cancers. A similar incidence was found in inflammatory breast cancers from Tunisia and in gestational breast cancers. Because these conditions represent highly invasive malignancies, it has been concluded that HMTV is sometimes associated with a particularly malignant phenotype [10].

The presence of a human mammary tumor virus (HMTV) has been reported with sequences [26] that are 90% to 98% homologous to MMTV, the etiological agent of mammary tumors in mice [15, 27]. It has been shown that the retrovirus is found in greater frequency in mammary tumors, and it is capable of integrating and becoming expressed in mammary tissue. The presence of MMTV or HMTV sequences has been investigated, but these sequences are not always found in normal breast tissue [11, 18, 24, 26, 28, 29]. For example, HMTV sequences have been found in 40% of breast cancers in both American and Australian women, whereas viral sequences were detected only in 1% of the non-affected mammary tissues from the same patients [11].

The association of an HMTV agent with human breast cancer remains controversial. The aim of this work was to evaluate the presence and current prevalence of the retrovirus HMTV (MMTV-like) sequences in BC in Mexican women.

## Methods

### Patients and tissue samples

Breast tissue samples from 458 Mexican women were collected between 2010 and 2013 at the Oncology Services of the Oncology Hospital, National Medical Center S-XXI, IMSS; Gynecology and Obstetrics Hospital No. 3. National Medical Center “La Raza”, IMSS; Oncology and Gynecology Hospital No. 4 Luis Castelazo Ayala, IMSS; and the National Cancer Institute, SSA, Mexico City Mexico. The histopathological diagnosis of each tumor was performed according to the World Health Organization criteria [30]. Samples were collected in separated containers with dissection material (hypochlorite 5%, and sodium hydroxide 5 N for 1 hour) prepared for each one and type of tissue (Tumor or normal breast tissue), and for each procedure: pathology, surgery or transport, to avoid any possible contamination from viral DNA or cells.

Cases were selected and matched with adjacent normal breast tissue (at least 2 cm and far from the tumor lesion) obtained at the time of the primary surgery. The sample was frozen in liquid nitrogen and stored at -80°C until use. Specimens were selected for analysis based on two criteria: (i) the presence of sufficient material for analysis and (ii) histological evaluation by two pathologists that demonstrates that the samples contained at least 30% tumor cells (in the case of the cancer samples). Representative sections from each case were paraffin-embedded for staining with hematoxylin and eosin to assess the histopathology diagnosis using the American Cancer Committee criteria.

The design was a cross-sectional prospective study. The study was approved by the Ethical Committee from the National Scientific Research Commission of the IMSS under the registration number 2008-785-067. Support data are available through the LabArchives repository with Accession Number DOI 10.6070/H47H1GHP. All of the data were maintained under strict confidentiality and complied with the National and International Regulations for health research in humans. All of the experiments were conducted in accordance with the Declaration of Helsinki, and all of the subjects exhibited an adequate understanding of and provided written consent for all of the procedures.

#### DNA extraction

The genomic DNA from frozen tumor and corresponding normal breast tissues was extracted using TissueLyser (Qiagen) for 20 seconds. The total DNA of each sample was obtained from 10–50 mg of tissue using a DNeasy Blood and Tissue Kit (Qiagen) with an automated QIAcube (Qiagen) according to the manufacturer’s instructions. The quantity and quality of the DNA determined through spectrophotometry by measuring the 260/280 ratio (NanoDrop, Thermo Scientific). We increased the amount of starting DNA from the samples that yielded low amounts of DNA using a WGA1 kit (Sigma-Aldrich) following the manufacturer's instructions. The DNA was purified with a GenElute PCR Clean-Up Kit (Sigma-Aldrich) and frozen at -80°C until use. The DNA integrity was evaluated by amplifying a 700-bp sequence of the *GAPDH* gene (GenBank, Gene ID 2597, glyceraldehyde 3-phosphate dehydrogenase) [31] followed by agarose electrophoresis. All of the PCR reagents were obtained from Life Technologies Invitrogen.

### Detection of HMTV (MMTV-like) sequence by PCR

The detection of HMTV *env* gene-like sequences was performed via nested PCR using the methods described by Wang *et al.* [32]. Specifically, a 660-bp fragment was amplified from the *env* gene of HMTV (MMTV-like) using primers 1 (5'-CCTCACTGCCAGATC-3') and 4 (5'-GAATCGCTTGGCTCG-3') with 200 ng of DNA as the template. The second PCR step was performed with primers 2 N (5'-TACATCTGCCTGTGTTAC-3') and 3 N (5'-ATCTGTGGCATACCT-3’), resulting in a 250-bp PCR product. The PCR products were analyzed by electrophoresis on 2.0% agarose gels stained with SyberGold 50X. As a negative control, nuclease-free sterile water was reacted with the primers in the absence of a template. The pBR322 vector (Cat 45006, ATCC) containing the MMTV *env g*ene from the C3H strain (GenBank AF228552) and propagated in *Escherichia coli* XL1 Blue [33] was used as the positive control. All of the procedures were carried out in a laminar flow hood exclusively used for PCR reactions with human DNA.

### Tests for mouse DNA and plasmid contamination

The Intracisternal A-type particle (IAP) PCR detection to discard mouse DNA contamination was performed with primers forward (5'- ATAATCTGCGCATGAGCCAAGG-3') and reverse (5'- AGGAAGAACACCACAGACCAGA-3’), resulting in product of variable size between 200 and 300-bp, reflecting the diversity of the IAP. The PCR products were analyzed by electrophoresis on 2.0% agarose gels stained with GelRed 10X. As a negative control, nuclease-free sterile water was reacted with the primers in the absence of a template. Mouse DNA was used as the positive control. Using the methods described by Oakes *et al.* [34].

The PCR detection to discard plasmid contamination was performed with primers TetCF (5'- AACAATGCGCTCATCGT-3') and TetCR (5'- GGAGGCAGACAAGGTAT-3’), resulting in a 1138-bp PCR product. The PCR products were analyzed by electrophoresis on 2.0% agarose gels stained with GelRed 10X. As a negative control, nuclease-free sterile water was reacted with the primers in the absence of a template. The pBR322 vector (Cat 45006, ATCC) containing the MMTV *env g*ene from the C3H strain (GenBank AF228552) and propagated in *Escherichia coli* XL1 Blue [33] was used as the positive control. Using the methods described by Lucarelli *et al.* [35].

Support data are available through the LabArchives repository with Accession Number DOI 10.6070/H4GH9FVX

### Real-time PCR

To identify the samples positive for the HMTV (MMTV-like) *env* gene, we designed primers and a TaqMan probe based on the reported sequences of the full C3H MMTV and human genomes (GenBank AF033807 and AF346816, respectively) as follows: forward 5'-AAGGGTGATAAAAGGCGTATGTG-3', location 5943–5964; reverse 5'- TTTTGTATTGGCCCCTGAGTTC, location 5990–6011 -3'; and probe 5'-FAM-AACTTTGGTTGACTACCTT-MGB-3', location 5969–5986. The reaction contained 100 ng of DNA, 900 nM of each primer, 250 nM probe, and the TaqMan Universal Master Mix II (Applied Biosystems). The reaction was performed according to the manufacturer’s instructions in a StepOne device (Applied Biosystems) with 60 cycles (95°C for 15 min and 60°C for 60 min). As a negative control, nuclease-free sterile water was reacted with the primers in the absence of a template, and the MMTV *env* gene from the C3H strain (GenBank AF228552) was used as a positive control. To calculate the sensitivity of the test and the viral copy number, we generated a complete standard curve with the plasmid DNA from the MMTV C3H strain by preparing serial dilutions (10^-1^ – 10^-7^) for the positive control reaction with 1 ng of template. All of the reactions were performed in duplicate.

### DNA sequencing

To confirm the presence of viral DNA and to rule out the amplification of non-specific or endogenous retroviral sequences, the PCR products of selected positive BC samples were sequenced. For this process, the nested PCR products (250 bp) were removed from the gel, purified using a Wizard SV gel and PCR Clean-up system (A9282 Promega Corp., Madison. USA), and submitted for sequencing to the Sequencing Unit facilities at the Molecular Biochemistry Department, UBIPRO, FES-Iztacala, UNAM. All PCR products were sequenced with an automatic capillary sequencer using the Sanger method with fluorescent terminators (ABI 3100). Support data are available through the LabArchives repository with the following accession numbers for the unaffected and tumor sequences: DOI 10.6070/H4MS3QQ6 and DOI 10.6070/H4C8277D, respectively. The sequences were compared with GenBank (AF033807, AF228552 and AF346816). The identity with HMTV and MMTV was determined using GeneFisher 2. We compared the sequencing data using the CLC Genomics Workbench 7.0.4 analysis software. We aligned all of the sequences using CLC Genomic Workbench software with the following parameters: gap open cost, 10; gap extension cost, 1; and alignment type, very accurate. After alignment, we constructed a tree using the Neighbor Joining Method with the Jukes-Cantor nucleotide distance measure and performed bootstrap analysis with 100 replicates.

## Statistical analysis

Descriptive statistics were used for the analysis of each of the study variables. The association between the presence of MMTV-like sequences in tumors and some sociodemographic and clinic data (age and type of tumor) was determined. The *X*^2^ test was appropriate for these analyses. *P* values of less than 0.05 were considered statistically significant. All of the analyses were performed using the SPSS 20 statistical software package.

## Results

### Breast tissue samples

The presence of HMTV (MMTV-like) sequences in tumor tissues and corresponding normal breast tissues collected from 458 Mexican women with breast cancer was determined. The tumor tissues were obtained from patients with a mean age of 56 years (range 25–89 years), and all of the samples were of sporadic tumors that had not been treated. Of the 458 patients, 13 patients (2.84%) had ductal carcinoma *in situ*, 301 patients (65.72%) had infiltrating ductal carcinoma, 58 patients (12.66%) had lobular carcinoma, 30 patients (6.55%) had mixed carcinoma (ductal-lobular), 12 patients (2.62%) had breast fibroadenoma, 27 patients (5.9%) had some other type of tumor, and there was no information provided for 17 patients (3.71%).

### HMTV detection by nested PCR

**Figure 1:**
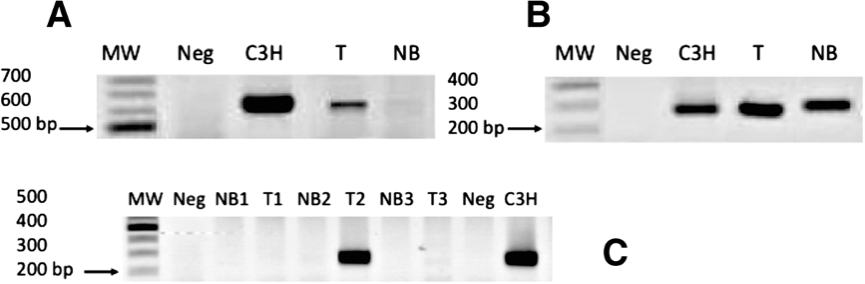
**End-point PCRs for detection of the MMTV** ***env g*** **ene in the samples. A**. First nested PCR round resulting in a 660-bp MMTV *env g*ene fragment, exemplifying the expected results defining positive detection. **B**. Second nested PCR round resulting in a 250-bp fragment, exemplifying the expected results defining positive detection. **C**. Three samples that exemplify positive and negative detection of the 250-bp PCR product. MW: 100-bp molecular weight ladder; Neg: non-template negative control; C3H: strain with MMTV *env g*ene inserted into pBR322 plasmid, used as a positive control; T: tumor; and NB: unaffected tissue.

The integrity of all of the DNA samples was validated by PCR amplification of a 600-bp fragment of the *GAPDH* gene. We detected HMTV (MMTV-like) through PCR amplification of the *env* gene (Figure 1). Figure 1-A presents the 660-bp fragment corresponding to the positive samples and controls obtained from the first PCR, and Figure 1-B shows the fragment corresponding to the 250-bp band in the positive samples and controls, which confirmed the presence of the amplified *env* gene fragment. Figure 1-C shows the three patients in whom the presence of HMTV was detected. Through the nested PCR analysis, HMTV sequences were detected in 129 samples (14.08%) of the 916 DNA samples from tumor and adjacent tissues. The prevalence of HMTV in the tumor tissues was 12.4%, and that in the non-affected breast tissues was 15.7%. In addition, the prevalence of HMTV in both the tumor and adjacent tissues from the same patient was 8.3%. An increased frequency of the virus was observed in patients aged 46–55 years. There was no significant difference between the tumor and adjacent tissue groups. The most frequent histopathological type was infiltrating ductal carcinoma (Table 1). Also all positive samples were checked to discard plasmid and mouse DNA contamination, accordingly to methods (data not Show).

**Table 1:** **Prevalence of HMTV by sex, age and type of tumor**

Prevalence %	Tumor and normal breast tissues	Tumor	Normal breast tissues	***P***value
Variables	(n = 38/458)	(n = 57/458)	(n = 72/458)	
	8.3	12.4	15.7	
**Age (years)**				
25-35	1(2.6%)	3(5.3%)	1(1.4%)	0.129
36-45	7(8.4%)	12(21.1%)	12(16.7%)	
46-55	15(39.5%)	21(36.8%)	21(29.2%)	
56-65	11(28.9%)	13(22.8%)	29(40.3%)	
66-75	4(10.5%)	6(10.5%)	8(11.1%9	
76+	0	2(3.5%)	1(1.4%)	
*P* value	0.129			
**Histopathological type**				
Ductal carcinoma *in situ*	1(2.6%)	1(1.8%)	1(1.4%)	0.271
Infiltrating ductal carcinoma	28(73.7%)	41(71.9%)	56(77.8%)	
Lobular carcinoma	6(15.8%)	9(15.8%)	12(16.7%)	
Mixed carcinoma (ductal + lobular)	1(2.6%)	1(1.8%)	1(1.4%)	
Breast fibroadenoma	0	0	0	
No sufficient data	0	1(1.8%)	0	
Others	2(5.3%)	**4(7.0%)**	2(8.3%)	
*P* value	0.356			

P < 0.001 was considered significant.

The cancer type classification was based on international criteria.

#### Verification of real-time PCR-positive samples

**Figure 2:**
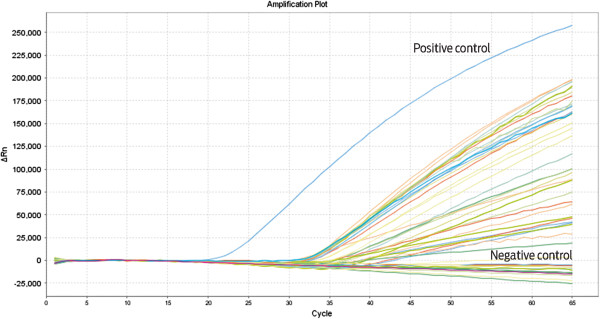
**Real-time PCR.** The plot shows the amplification obtained using TaqMan probes with the same DNA sources as those used for the nested PCR that yielded positive results. The probes are based in a different region of the *env* gene, namely from 5943 to 6011 (AF033807). Each line represents a sample, positive control, or negative control. The PCR cycles are shown on the x-axis, and the ΔRn values (representing the fluorescent units) are shown on the y-axis. All of the assays were performed in 48-well plates.

We performed real-time PCR with TaqMan probes corresponding to a different region of the *env* gene than the region that was used for the nested PCR; however, we used the same source of DNA, a 68-bp fragment located from 5943 to 6011 (AF033807). Figure 2 shows the amplification curves, which demonstrate that most of the positive samples (96/129 positive samples; 74.41%), as well as the negative and positive control, could be verified by this method. Of the 129 positive samples detected by nested PCR, 33 (25.58%) could not be verified with the TaqMan probes.

#### Sequence alignment and tree construction

**Figure 3:**
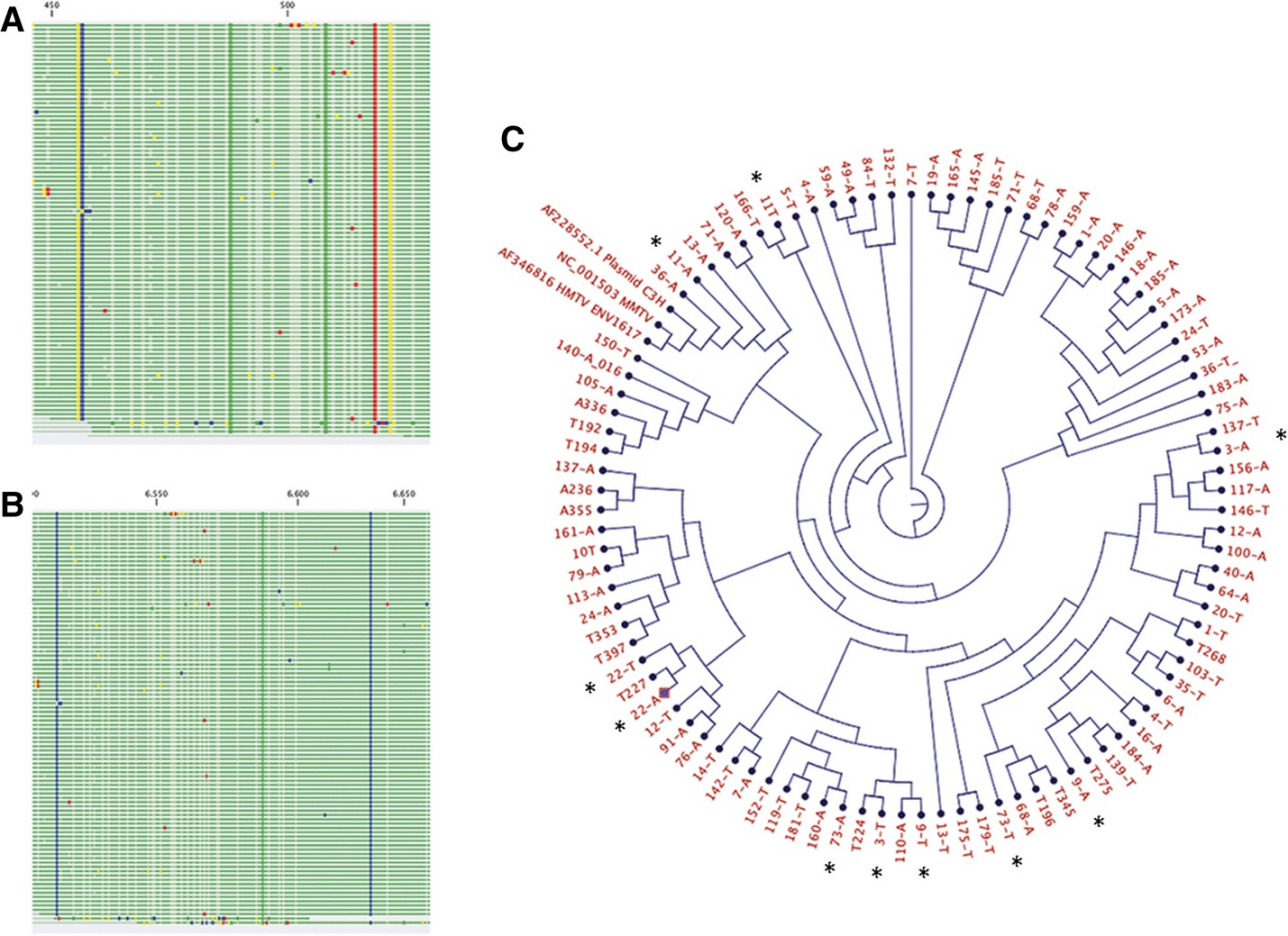
**Alignment and tree. A**. Alignment of 96 samples against the MMTV sequence (AF033807). **B**. Alignment of 96 sample sequences against the HMTV sequence (AF346816). **C**. Tree constructed by the neighbor-joining method. The tree contains 96 positive samples and depicts the relationship among them, MMTV, HMTV and C3H.

To determine and verify the identity of the positive samples, 96 samples (breast tumors and adjacent tissues) were randomly selected. The PCR fragments were isolated from the agarose gels and purified, and the DNA was sequenced. Figure 3A shows the sequences of amplified fragments. The sequences were aligned to the corresponding region of the reported MMTV and HMTV sequences. The comparison of MMTV with the consensus sequence revealed a percentage of similarity of 99%. We also found at least three bases in all sequenced samples that were different from those in the reported MMTV sequence. The comparison of HMTV with the consensus sequence resulted in a percentage of similarity equal to 98%. As shown in Figure 3B, at least six bases are different from those in the reported HMTV sequence, and these bases are present in all of the sequenced samples. The individual sequence difference presented up to 88.8% homology with the reported sequences.

We then performed a simultaneous alignment using the 96 sequenced samples, MMTV, HMTV, and C3H strain to obtain a tree using the neighbor-joining method, which is shown in Figure 3C. The tree forms three main branches and shows the relationship between the samples and the reported sequences for MMTV, HMTV and C3H. We noted that the samples from the same patients (tumor and unaffected tissues) remained near or not too far from their corresponding matched samples, as exemplified by the patient sample pairs 3A and 3 T, 9A and 9 T, 11A and 11 T, 22-A and 22 T, and 73A and 73 T, and that MMTV and HMTV tended to be in different groups or in different branches.

## Discussion

Since Bittner described the mouse mammary tumor virus (MMTV) as the agent involved in mouse mammary carcinogenesis in 1936, MMTV has been regarded as a potential model for human disease. The presence or absence of MMTV-like virus in breast cancer samples from several populations has been reported in the last five decades. The presence of the virus has been measured by sequencing and immunoreactivity against the envelope protein (Env), and retroviral particles have even been found in normal human milk samples [10].

The current technology and the developed methodologies allow us to distinguish exogenous sequences that are not present in the human genome that are 95% to 99% homologous to MMTV [32]. A low prevalence or absence of the viral sequences has been reported in normal tissue, ruling out the germline transmission of or polymorphisms in endogenous retroviral sequences [28].

Since the beginning of this century, this retrovirus has been designated a human mammary tumor virus (HMTV) [27]. In this study, we found an HMTV prevalence of 12.4% in breast cancer samples, with a higher prevalence among women aged 46–55 years, which is similar to the prevalence reported in other studies. In the Mexican population (northeastern Mestizo), the prevalence of MMTV-like sequences in breast cancer patients has been reported to be 4.2% (n = 119) [24]. We decided to increase the size of the population (n = 458) and to determine the presence of HMTV in breast cancer *vs.* normal breast tissues within the same patient. Surprisingly, we observed a 15.7% prevalence of HMTV in normal breast tissue, with a higher prevalence among women aged 56–65 years. This prevalence was higher than that found in previous studies. The presence of the retrovirus in breast tumor and normal breast tissue samples from an individual patient presented a prevalence of 8.3%. We confirmed and extended previous findings showing that the differences in the prevalence of HMTV (MMTV-like) gene sequences and breast cancer rates among populations may be related to the geographic distribution [22].

We agree with the model that HMTV is not passed down through the germ line because it would then be present in all cells of a given patient, although this possibility remains a latent question regarding transmission in humans. However, this model raises a question regarding the increased presence of HMTV in normal breast tissue. We can dismiss the possibility of contamination because we strictly followed the appropriate technical methods, such as working in a fume hood exclusively used for human DNA. In addition, our staff never worked with murine lines or genetic material, and the positive controls were prepared separately. It has been proven by this method that the *env* gene is not present in humans and is different than HERVs (human endogenous retroviruses) [10]. We checked all samples to discard contamination to avoid false positives from mouse DNA or plasmid, corroborating the correct samples management. Supporting our present results, in which normal cells even far from the tumor or near, are infected but they are not yet transformed.

Therefore, we verified the HMTV positivity using two distinct methods. We designed new primers and a TaqMan probe for a different region 36 bp from the fragment amplified by the nested method. These primers were based on the same reported sequence, and we expected to corroborate all of the positive results obtained by nested PCR. However, we could only verify the results found for 74.41% (96/129) of the samples that were found to be positive by nested PCR, as well the controls, and randomly selected patient samples that had been found to be negative by nested PCR patient samples. We assume that this difference is due to the fact that was because the retrovirus has a high rate of mutation, with a reported homology of 90% to 98% [9, 10, 23]. In addition, it is known that the TaqMan systems are specific and sensitive, and they do not accept base changes.

To determine the identity of the amplified samples, we sequenced the 250-bp fragment from 96 positive samples by the Sanger method. The sequences were aligned and compared with the reported references for MMTV and HMTV. Because the sequences are not very long, it is difficult to establish the presence of the complete virus and to determine whether the sequence varies even within the same patient. However, with the generated data, we could appreciate that, even though the *Env* gene is a conserved sequence, we did find polymorphism among the samples with up to 88.8% homology. This finding also corroborates the lack of contamination and also highlights differences between the reported sequences for MMTV and HMTV and the consensus sequence, which revealed three to six different bases. Our results also support the contention that these sequences may represent a virus associated with human breast carcinogenesis [22]. The analysis suggests that there are two different viruses. The similarity between the sequences indicates that they are closely related but not identical.

The presence of HMTV gene sequences may contribute to the development or progression of carcinogenesis. Thirty-eight of the evaluated patients had both tumor and unaffected tissues that presented positive results, and we observed similarity among the amplified sequences in the constructed tree due to the fact that they were positioned in the same branch and had similar homology to one another. This observation may indicate two independent infections or an infection of common origin with or without a mutation in HMTV. The mechanism through which MMTV may contribute to breast cancer in mice is known: the virus reaches the cell, the RNA is converted to DNA, and the DNA is integrated into the host genome in certain specific locations. It has also been shown that the envelope gene of MMTV alone can cause murine cells and human mammary cells to become cancerous, and the overexpression of the envelope gene may be the cause of certain cancers [8]. The capsid is one of the proteins on the outside of the virus particle, and it is believed that this protein connects the virus to the host cell receptor and likely helps it penetrate into cells. It is known that the envelope contains ITAM (immunoreceptor activation motif based on tyrosine), which stimulates certain hormones and is suggested to contribute to the initiation of breast cancer. Env and ITAM proteins in human breast cancer are likely oncoproteins [36]. The proviral structure is replication-competent, and its long terminal repeat [3] contains several hormone-responsive elements and the open reading frame for superantigen [10, 37]. Regarding HMTV and human non-affected mammary cells, there is strong evidence of the presence of HMTV in human breast cancer, but the receptor that is used to penetrate into the cell is not know, and mechanism that allows the virus to infect the host is not clearly understood. In addition, we hypothesize that the virus effect may depend on the site of insertion of the retrovirus. In 2007, Theodorou *et al.* performed a high-throughput retroviral insertional mutagenesis screen in mouse mammary tumor virus (MMTV)-induced mammary tumors and identified 33 common insertion sites, of which 17 were previously not known to be associated with mammary cancer and 13 had not been previously linked to cancer in general [38]. Consequently, there should be differences among breast cancer samples and unaffected tissues related to the presence of HMTV and the sites of insertion. If the retrovirus gets inserted into a region that activates an oncogene or inactivates a tumor suppressor or repair gene, the insertion may alter signaling pathways and contribute to tumor development or progression by insertional mutagenesis. In addition, insertion may simply trigger the neoplastic process that is waiting to start. In contrast, if the retrovirus inserts into a site with no significant biological function (due to the large amount of LTRs along the human genome), the cell will not be damaged, and the presence of viral DNA will simply become a risk factor, as in the case of infected normal tissue. We would like to reinforce that viruses in humans are acquired and should not be considered a HERV. Moreover, the insertional mutagenesis caused by HMTV may explain the heterogeneity found in breast cancer tumors. This heterogeneity limits an accurate response to treatment because the infection may occur before or after neoplastic transformation. According to the present data, it is necessary to look deeper for the mechanism of infection of this retrovirus in humans, to determine the contribution of HMTV to the development and progression of breast cancer (or any neoplasia), to identify the sites of insertion and define the mutagenesis that insertion may cause, to detect the presence of the virus in serum for diagnostic purposes, to prevent the infection by identifying and therapeutically blocking the receptor, and to correlate infection with tumor aggressiveness when infection is present. We are currently subjecting the positive samples to NGS (next-generation sequencing) in order to identify the insertion sites in our samples, which would allow us to discard HERVs from new insertions. We hypothesize that this strategy will help us investigate whether the presence of HMTV in women and in the mammary gland is a risk factor for oncogenesis. We also hypothesize that this strategy will contribute to the determination of whether HMTV provokes viral oncogenesis and whether it is indeed a prominent causative agent of breast cancer. As was shown recently, the importance of identifying the heterogeneity at the molecular and genomic levels will provide important data for studying specific types of human breast cancer [39]. Understanding retroviral behavior will contribute to the development of immunological therapies against cancer, and further research will aid the diagnosis, treatment and prevention of cancer caused by viruses [40].

## Conclusions

These results confirm that the HMTV sequence is present in the Mexican population, in both breast tumors and unaffected tissue. There is strong evidence supporting the presence of HMTV in human breast cancer, which reinforces the conclusion that viruses in humans are acquired and should be considered if HMTV is indeed a prominent causative agent of breast cancer. Insertional mutagenesis caused by HMTV may explain the heterogeneity found in breast cancer tumors and its innocuous presence in unaffected mammary gland tissue in women.

## Abbreviations

A: Adjacent
BC: Breast cancer
DOI: Digital object identifier
Env: Envelope protein
*env*: Envelope gene
*GAPDH*: Glyceraldehyde 3-phosphate dehydrogenase
HERVs: Human endogenous retroviruses
HMTV: Human mammary tumor virus
IAP: Intracisternal A-type particle
ITAM: Immunoreceptor activation motif based on Tyrosine
LTR: Long terminal repeat
MMTV: Mouse mammary tumor virus
NGS: Next-generation sequencing
Sag: Superantigen
T: Tumor.
